# Probiotic Spore Inspired Oral Drug Delivery System With Multiple‐Barriers‐Crossing Ability Modulates Gut‐Brain Axis for Parkinson's Disease Therapy

**DOI:** 10.1002/exp2.70221

**Published:** 2026-09-09

**Authors:** Qianhua Feng, Yuying Mei, Jia Zhang, Liying Sun, Bingqian Si, Huimin Zhou, Huifang Xiao, Runhan Liu, Jieyun Zhang, Lei Wang

**Affiliations:** ^1^ School of Pharmaceutical Sciences State Key Laboratory of Antiviral Drugs Pingyuan Laboratory Zhengzhou University Zhengzhou China; ^2^ Henan Key Laboratory of Targeting Therapy and Diagnosis for Critical Diseases Zhengzhou China; ^3^ Department of Pharmacy Henan General Hospital Zhengzhou China; ^4^ Hubei Provincial Clinical Research Center for Central Nervous System Repair and Functional Reconstruction Taihe Hospital Hubei University of Medicine Shiyan Hubei China; ^5^ School of Electrical Mechanical and Biomedical Engineering Faculty of Engineering and Information Technology University of Technology Sydney Sydney New South Wales Australia

**Keywords:** microbiome‐gut‐brain axis, Parkinson's disease, probiotic spores

## Abstract

For Parkinson's disease (PD), the development of oral brain‐targeted drug delivery is challenging due to various physiological barriers. Besides the brain, the effect of the gut on the microbiome‐gut‐brain axis should not be ignored. Therefore, developing a gut‐brain dual‐targeted delivery system with multiple‐barriers‐crossing ability is meaningful. During the germination of probiotic spores, we find that spore coat proteins (SP) shed from their surface possess excellent characteristics (re‐assembling into nanoparticles, multiple‐barriers penetration ability, antioxidant property, etc.), offering potential for brain‐targeted drug delivery. Inspired by this, we propose a probiotic spore based oral drug delivery system to dual‐regulate the gut‐brain. After oral administration, the spore system crosses the harsh gastrointestinal chemical barrier. Spores germinate in the intestine into probiotics to modulate the gut‐brain axis. Meanwhile, the SP‐derived reassembled nanosystems cross multiple barriers (intestinal mucus barrier, enterocyte barrier, blood‐brain barrier) to reach brain and further reduce oxidative stress. Notably, neurodegeneration and behavioral deficits are alleviated in PD mice. Such gut‐brain dual‐regulation strategy emphasizes a comprehensive and promising technique for central nervous system disease treatment, which may be an alternative to traditional single‐targeted methods. Furthermore, the probiotic spore‐based oral biotherapeutics with multiple‐barriers‐crossing efficiency provide new insight into other brain diseases.

## Graphical abstract

Inspired by the germination of probiotic spores, we utilize the excellent characteristics of spores to break through multiple barriers of oral brain‐targeted delivery. Besides the probiotics mediated gut‐brain communication, the spore coat proteins derived nanosystems with multiple‐barriers‐crossing ability alleviate neurodegeneration. This work emphasizes a comprehensive technique for gut‐brain dual‐regulation in Parkinson's disease treatment, which may be an alternative to traditional single‐targeted method.

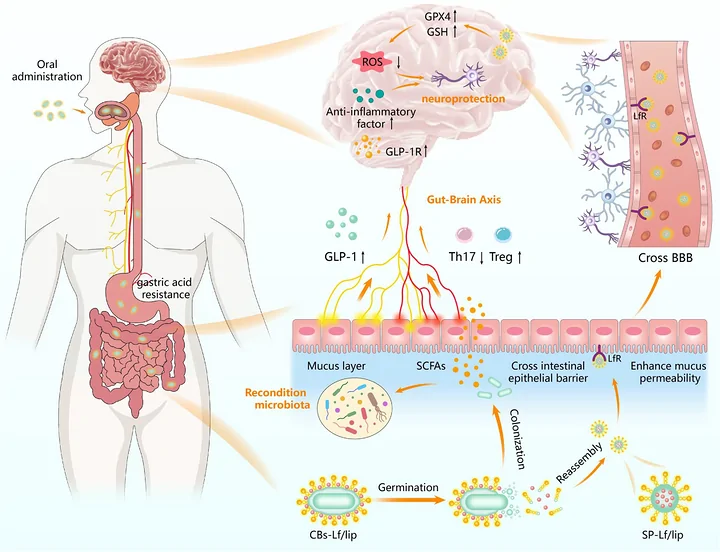

## 1 Introduction

Parkinson's disease (PD), as a common neurodegenerative disease, is characterized by motor dysfunction and memory impairment due to dopaminergic neurons loss [1, 2]. At present, the clinical treatments mainly rely on dopaminergic replacement therapy (levodopa, dopamine receptor agonists, etc.) [3]. Unfortunately, the lack of effective neuroprotection makes this method difficult to inhibit or reverse the progress of PD, which implies the requirement of developing novel strategies. Previous studies have indicated that oxidative stress and neuroinflammation constitute risk factors for neurodegeneration [4]. Some designed nanoplatforms which deliver antioxidants and anti‐inflammatory agents to the brain become promising, however, they are mainly administered intravenously or intraventricularly with poor compliance [5, 6]. Despite its advantages such as convenience and safety, noninvasive oral brain‐targeted drug delivery is studied sparsely because of inevitable multiple barriers: (I) chemical barrier (e.g., gastric acid, bile salt), (II) intestinal mucus barrier, (III) enterocyte barrier, (IV) blood–brain barrier (BBB) [7, 8]. Therefore, designing an oral anti‐PD system that could overcome various barriers of oral delivery for high bioavailability poses a challenging yet crucial avenue of research.

With the in‐depth study of the microbiome‐gut‐brain axis, the activity of gut microbes is closely related to brain function, as evidenced by gut microbiota dysbiosis and gastrointestinal symptoms in the early stage of PD [9, 10]. Recently, probiotics‐based oral live biotherapeutics mediate gut‐brain communication, providing new insights for PD treatment [11]. Notably, *Clostridium butyricum*, known as the intestinal guard, reshapes gut microbiota and improves the intestinal environment [12]. These probiotics secrete neuro‐active metabolites such as short‐chain fatty acids (SCFAs) to restore intestinal immune homeostasis as well as promote intestinal glucagon‐like peptide‐1 (GLP‐1) secretion, thereby affecting neurophysiology and reducing neuroinflammation for neuroprotection through gut‐brain signaling (endocrine pathway, immune system, nerve pathway) [13, 14]. In this sense, for the treatment and especially prevention of PD, the gut as the “second brain” cannot be ignored, except for the primary target of the brain. Thus, it is highly urgent to devise an approach that possesses precise gut‐targeted probiotic delivery and brain‐targeted drug delivery with multiple‐barriers‐crossing ability, simultaneously modulating gut and brain microenvironment for PD treatment.

Spores, the dormant life form of some probiotics such as the *Bacillus* genus, can germinate into metabolically active probiotics in the intestine, which is becoming increasingly attractive in microbial therapy [15, 16, 17]. During the germination process, the hydrophobic spore coat proteins (SP) are shed from the surface of the spore and reassemble into nanoparticles as demonstrated by our previous research, offering the potential for oral brain‐targeted drug delivery [18]. More significantly, as a protective layer for spores, the SP with a durable static biological structure confers high resistance against a harsh gastrointestinal environment containing gastric acid, bile salts, and enzymes. In this study, on the basis of maintaining a high formation efficiency of the reassembled nanoparticles, we find that SP‐derived nanoparticles display intestinal mucus penetration performance, which could be ascribed to the sulfhydryl groups of cysteine abundant in SP undergoing thiol‐disulfide exchange with mucin to cleave the mucus network. Excitingly, benefiting from the sulfhydryl groups with antioxidant properties in SP, the antioxidant activity of SP is also confirmed for the first time in this work. Given the advantages of spores discovered above, the probiotic spore‐based delivery system brings hope for overcoming multiple barriers of oral brain‐targeted delivery and reducing oxidative stress in the brain, which also brings opportunity to the dual‐regulation of gut‐brain.

Inspired by the germination of probiotic spores, this study develops an oral anti‐PD drug delivery system using probiotic spores as a delivery carrier, aiming to precisely target and regulate the gut and brain with multiple‐barriers‐crossing ability (Figure 1). Briefly, the *C. butyricum* spores (CBs) are modified with lactoferrin (Lf) and loaded with lipid peroxidation inhibitor (liproxstatin‐1, lip). After oral administration, CBs‐Lf/lip cross the harsh gastrointestinal chemical barrier and germinate in the intestine, while the spore coat proteins (SP) shedding from the spores are reassembled into SP‐Lf/lip nanoparticles. Then SP‐Lf/lip can penetrate the intestinal mucus barrier with the help of SP and cross the enterocyte barrier and BBB via Lf‐receptor (LfR) mediated transcytosis, which enhances the drug accumulation in the brain significantly. On one hand, the reassembled SP‐Lf/lip nanoparticles overcome various barriers of oral delivery to target the brain (the primary target). The oxidative stress and neuroinflammation are alleviated due to efforts in many ways, such as the antioxidant activity of SP, the enhancement of glutathione peroxidase activity by Lf, and the inhibitory effect of lip on lipid peroxidation. On the other hand, the CBs germinate into *C. butyricum* and colonize the intestine (the secondary target) to modulate the gut‐brain axis. Results showed that the CBs‐Lf/lip protected against dopamine neuron loss and rescued behavioral deficits in mice. In conclusion, we propose a gut‐brain dual‐regulation strategy for PD treatment. To our knowledge, no effort has yet been made to overcome various barriers to oral brain‐targeted drug delivery by utilizing the natural characteristics of probiotic spores. This designed oral live biotherapeutic agent as a more comprehensive technique for gut‐brain dual‐regulation may be an alternative to traditional outdated and single‐targeted methods, which provides new insight for the treatment of central nervous system disease.

**FIGURE 1:**
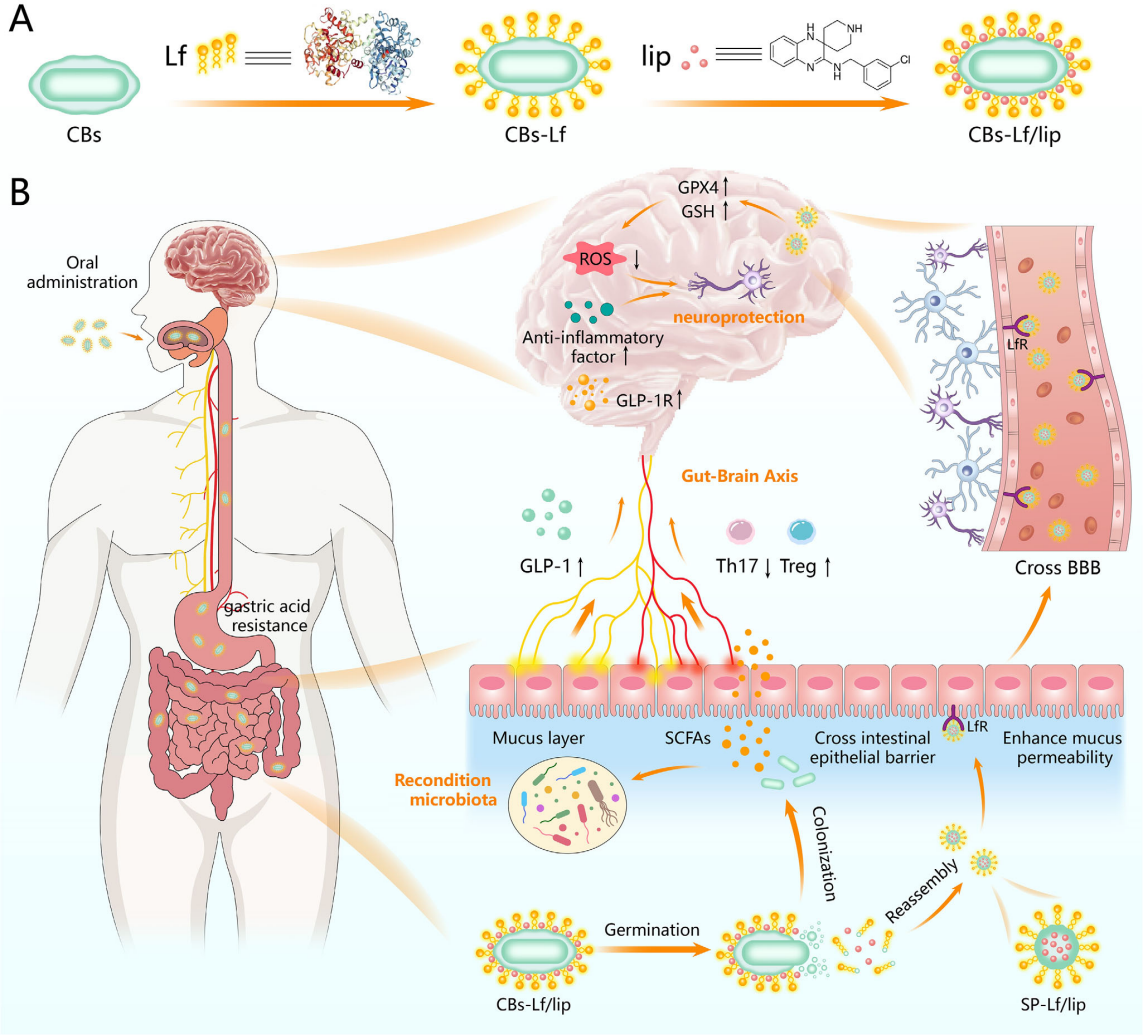
Design and mechanism of CBs‐Lf/lip for PD treatment. (A) The CBs are modified with Lf and loaded with lip to obtain CBs‐Lf/lip. (B) Schematic illustration of gut‐brain dual‐regulation. After oral administration, the CBs‐Lf/lip confer high resistance to a harsh gastrointestinal environment containing gastric acid. On the one hand, CBs germinate into *Clostridium butyricum* and colonize the intestine to reshape gut microbiota and secrete SCFAs. Via the gut‐brain axis, the SCFAs further restore intestinal immune homeostasis and promote intestinal GLP‐1 secretion, thereby affecting neurophysiology and reducing neuroinflammation. On the other hand, the spore coat proteins (SP) shed from spores are reassembled into SP‐Lf/lip nanoparticles, which penetrate the intestinal mucus barrier with the help of SP and cross the enterocyte barrier and BBB via LfR‐mediated transcytosis to reach the brain and reduce oxidative stress.

## 2 Results

### 2.1 Preparation and Characterization of CBs‐Lf/Lip

*C. butyricum*, which can secrete high levels of SCFAs, is known as the intestinal guard [12]. Herein, the *C. butyricum* spores (CBs) are selected as the drug delivery carrier in this work (Figure 2A). Both transmission electron microscopy (TEM) and scanning electron microscopy (SEM) showed their elliptical structure with a size of ≈2 µm (Figure 2B,C). Next, Lf was introduced onto the surface of CBs via an amidation reaction (Figure 2A). It seemed that CBs displayed a thin film‐like layer of Lf on their surface in TEM, while SEM images showed that there were granular substances attached to the surface of spores, which could be ascribed to the Lf modification. Then hydrophobic and positively charged liproxstatin‐1 (lip) was loaded onto the surface of spores with a high drug loading capacity of 32.1% via hydrophobic interaction and electrostatic adsorption. Compared with CBs‐Lf, the increased zeta potential of CBs‐Lf/lip and new emerged optical adsorption peak at 316 nm of lip indicated the successful lip loading (Figure 2D,E). The effects of Lf and lip on spore germination and growth were tested, as these were important for the feasibility of the modification. After culturing spores in growth medium, CBs germinated into *C. butyricum* and formed circular and creamy white colonies, as shown from colony plate images (Figure 2F). Compared with the CBs group, CBs‐Lf and CBs‐Lf/lip groups increased the colony counts (Figure 2G), which could be attributed to the fact that Lf acted as a prebiotic to promote the growth of *C. butyricum* [19]. In addition, OD600 detection showed similar growth profiles of *C. butyricum* (Figure S1). The above results revealed that the Lf modification and lip loading did not affect the activity of CBs, which provided conditions for the reassembly of SP during the germination of probiotic spores.

**FIGURE 2:**
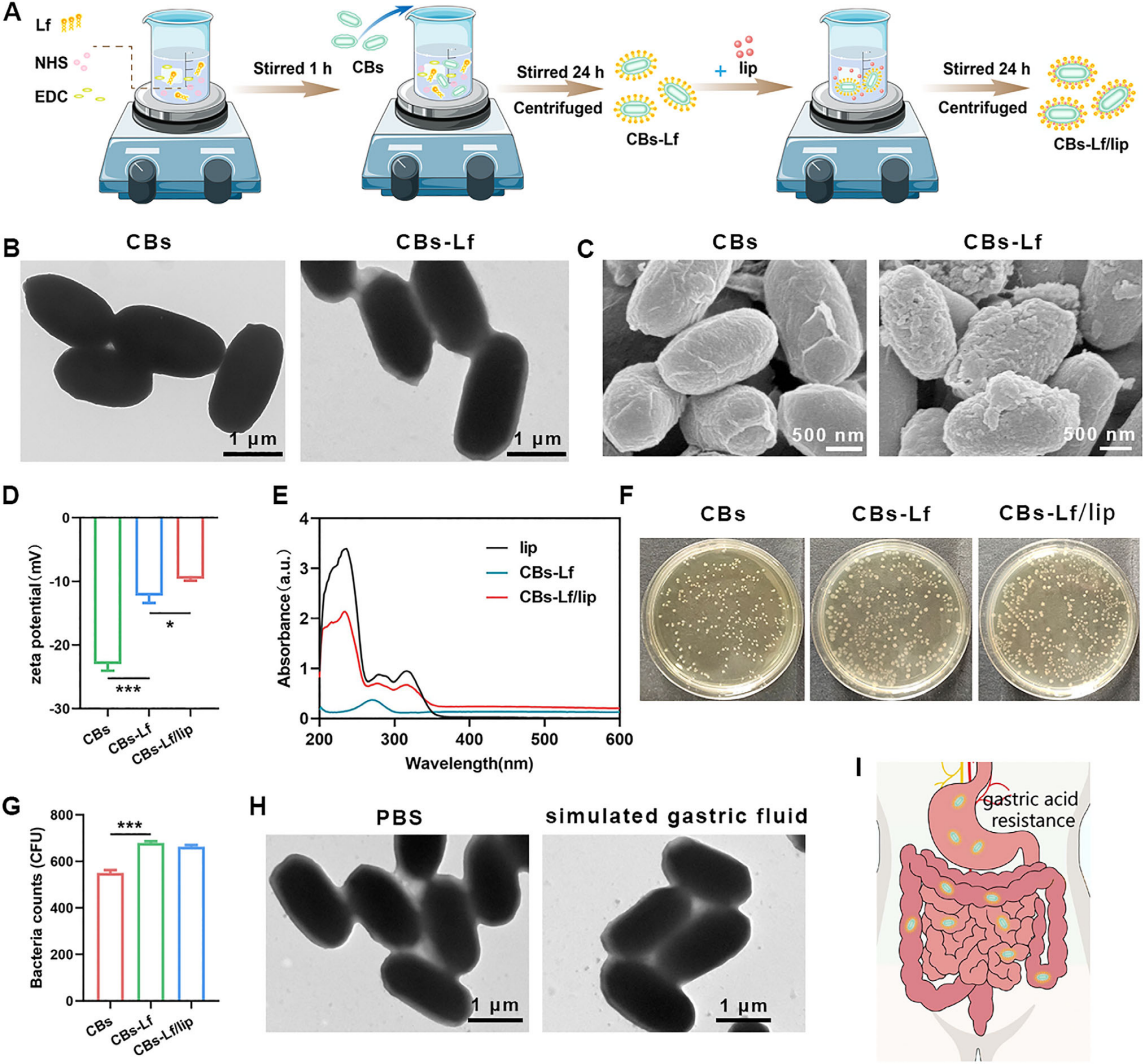
Characterization of CBs‐Lf/lip. (A) Schematic representation of the preparation of CBs‐Lf/lip. (B) TEM images of CBs and CBs‐Lf. (C) SEM images of CBs and CBs‐Lf. (D) Zeta potential analysis (*n *= 3). (E) UV–vis spectra. (F) Colony plate images. (G) The colony‐forming units (CFUs) statistics of bacteria (*n *= 3). The spores were spread onto an agar plate and incubated for 24 h before bacterial counting. (H) TEM images of CBs‐Lf/lip in PBS (pH = 7.4) and simulated gastric fluid (pH = 1.2). (I) Schematic overview of CBs‐Lf/lip's resistance to gastric acid. Data were presented as means ± SD. *P < 0.05, **P < 0.01, ***P < 0.001.

In reality, the application of probiotics‐based therapy encounters challenges such as the harsh gastrointestinal chemical barrier. The gastric acid, bile salts, or enzymes in the gastrointestinal region reduce the survival rate of probiotics and limit their colonization in the intestine [20, 21]. In contrast, spores, as the dormant life form of probiotics, are reported to confer high resistance against the external environment [22, 23]. Then, CBs‐Lf/lip were incubated in simulated gastric fluid (pH = 1.2) and PBS (pH = 7.4), respectively. TEM images showed there was no difference in morphology between the two groups, exhibiting the integral structure of spores (Figure 2H). Furthermore, the leakage rate of lip for CBs‐Lf/lip was measured to be only 14.49% within 2 h (Figure S2). These results indicated that CBs‐Lf/lip were capable of crossing the gastrointestinal chemical barrier (Figure 2I). Usually, SP with a durable static biological structure on the surface of the spore acts as a protective layer, which improves the stability and resistance of probiotics in the gastrointestinal region and reduces drug loss in gastric acid. Additionally, the stability of CBs‐Lf/lip after storage at 4°C for 14 days was evaluated. Figure S3 shows that CBs‐Lf/lip exhibited the integral structure of spores, implying good stability of CBs‐Lf/lip.

### 2.2 The Formation of SP‐Lf/Lip Nanoparticles

Our previous research has demonstrated that the deoxycholic acid‐modified SP shedding from the *Bacillus cagulan* spore could be reassembled into nanoparticles when the spore germinated in the intestine [18]. Herein, the feasibility of the reassembly behavior of SP on CBs‐Lf/lip was investigated. After incubation in growth medium for 24 h, CBs‐Lf germinated into *C. butyricum* with a straight or slightly curved structure (Figure 3A). It was worth noting that small spherical particles emerged in the surroundings of *C. butyricum*. Then these nanoparticles were separated from the growth medium and visualized by TEM and SEM; Figure 3B showed their uniform morphology with a particle size of ≈35 nm. When CBs‐Lf/lip germinate into probiotics in the intestine, the SP‐Lf protein and lip on their surface fall off. These Lf‐modified hydrophobic SP are able to self‐assemble into drug‐loaded nanoparticles (SP‐Lf/lip) (Figure 3C). The in vitro formation efficiency of the reassembled SP‐Lf/lip was as high as 76.3%. For the reassembly mechanism of the nanoparticles, previous research has merely hypothesized that hydrophobic interaction was involved. To further investigate the driving force of the reassembly, the SP‐Lf nanosystem was incubated with destructive agents for various forces (Figure S4). Results showed that the size of the nanosystem reduced obviously in the presence of Triton X‐100 (destructive agent of hydrophobic interaction), implying the disintegration of the nanosystem. The larger size of the SP‐Lf system in the presence of urea (destructive agent of hydrogen bonds) could be assigned to the fact that the SP‐Lf system disintegrated into small nanoparticles and then aggregated. The above results suggested that the reassembly of Lf‐modified hydrophobic SP was mainly driven by hydrophobic interaction and hydrogen bonds. Motivated by these results, we moved on to investigate the in vivo formation behavior of SP‐Lf/lip nanoparticles by bio‐TEM (Figure 3D). For the saline group, the intestinal villi of mice were arranged neatly, and no nanoparticles were observed in the intestine. However, the nanoparticles were observed in the intestinal loop and villi in the CBs‐Lf/lip group, indicating that nanoparticles were being taken up by intestinal epithelial cells. On the basis of our previous research on the reassembly of small molecule modified spores into nanoparticles, this study confirms that protein modified spores could still form nanoparticles in the intestinal environment, suggesting the reassembly is not limited by the modified materials and spore strains.

**FIGURE 3:**
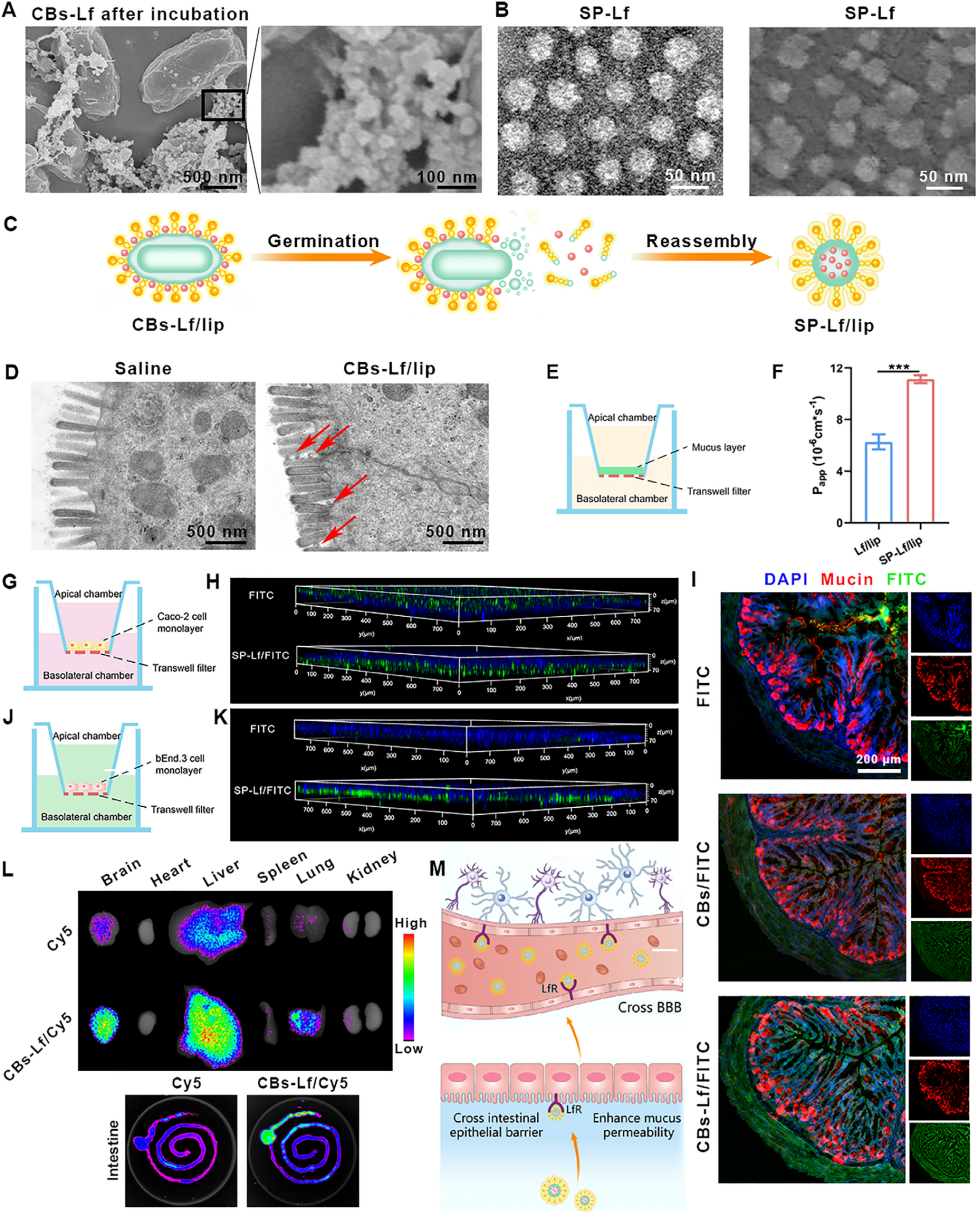
The formation and multiple‐barriers‐crossing ability of the reassembled SP‐Lf/lip. (A) SEM image of CBs‐Lf after incubation in growth medium for 24 h. (B) TEM (left) and SEM (right) images of nanoparticles (SP‐Lf) generated from CBs‐Lf after incubation in growth medium for 24 h. (C) Schematic diagram of the formation of SP‐Lf/lip. (D) Bio‐TEM images of intestinal tissue. The reassembled SP‐Lf nanoparticles were indicated by red arrows. (E) Schematic illustration of mucus model in vitro. Mucus layer is seeded in apical chamber. (F) The apparent permeability coefficient (*P*_app_) of Lf/lip and Sp‐Lf/lip (*n *= 3). (G) Schematic illustration of enterocyte barrier model in vitro. Human colon carcinoma Caco‐2 cells are seeded in the apical chamber. (H) 3D confocal images of FITC or SP‐Lf/FITC on Caco‐2 monolayer from apical to basolateral side. (I) Representative immunostaining micrographs showing FITC‐labeled formulations (green) penetrated the intestinal mucus (red) barrier and enterocyte barrier in vivo. (J) Schematic illustration of BBB model in vitro. Murine brain capillary endothelial bEnd.3 cells are seeded in the apical chamber. (K) 3D confocal images of FITC or SP‐Lf/FITC on bEnd.3 cell monolayer from apical to basolateral side. (L) Ex vivo fluorescence images of major tissues in mice after oral administration of Cy5 and CBs‐Lf/Cy5 at 24 h. (M) Schematic diagram of the multiple‐barriers‐crossing ability of the reassembled SP‐Lf/lip. Specifically, SP‐Lf/lip nanoparticles penetrate the intestinal mucus barrier with the help of SP, and cross the enterocyte barrier and BBB via LfR‐mediated transcytosis to reach the brain. Data were presented as means ± SD. *P < 0.05, **P < 0.01, ***P < 0.001.

### 2.3 The Multiple‐Barriers‐Crossing Ability of SP‐Lf/Lip

The development of oral brain‐targeted drug delivery is challenging and rarely attempted due to various physiological barriers such as the chemical barrier, intestinal mucus barrier, enterocyte barrier, and BBB. For the intestinal mucus barrier, the small size of nanoparticles (35 nm) is sufficient to allow their diffusion through the mucin fiber network with small mesh sizes (50–1800 nm) [24]. For the enterocyte barrier, numerous studies have shown that nanoparticles in the size range of 30–200 nm have the potential to penetrate the enterocyte barrier. Among them, nanoparticles with a size of 30–50 nm can penetrate intestinal epithelial cells with higher efficiency than larger‐sized nanoparticles [25]. As for the BBB, smaller‐sized nanoparticles exhibit higher BBB penetration ability. For example, gold nanoparticles with sizes of 30–40 nm show higher transport efficiency than nanoparticles with sizes of 50–60 and 70–80 nm [26]. In conclusion, the SP‐Lf/lip nanosystem with 35 nm is suitable for crossing multiple barriers in vivo to reach the brain.

The mucin, as the main functional component of the intestinal mucus barrier, can form a mucin fiber network through disulfide bonds, which capture and remove foreign objects to reduce the bioavailability of oral drugs [27]. Herein, the mucus penetration performances of SP‐Lf/lip and pre‐prepared lip‐loaded Lf nanoparticles (Lf/lip) were investigated in a transwell model of mucus (Figure 3E). The apparent permeability coefficient (*P*_app_) of SP‐Lf/lip was 1.8‐times higher than that of Lf/lip group (Figure 3F). The excellent mucus penetration ability of reassembled nanoparticles was closely related to their surface properties and particle size [28]. The cysteine with a sulfhydryl group was abundant in SP, and the content of sulfhydryl group in SP was as high as 12.8 µmol g^−1^ by using a sulfhydryl assay kit, which would undergo thiol‐disulfide exchange with mucin to cleave the mucus network. Furthermore, the negative charge of SP‐Lf nanoparticles forms electrostatic repulsion with the mucus layer to avoid a mucus trap, and the small size of the nanoparticle (35 nm) enhanced their diffusion through mucin fiber network with small mesh sizes (50–1800 nm).

Lactoferrin (Lf) is a multifunctional protein that provides nutrition, regulates immunity, and participates in signal transduction. Lactoferrin receptors (LfR) have a diverse family (more than 10 types). Among them, low‐density lipoprotein receptor‐associated protein 1 (LRP1) is a key receptor mediating Lf endocytosis [29, 30]. It was widely expressed in enterocytes and brain microvascular endothelial cells (Figure S5), which provided evidence supporting the transcytosis of CBs‐Lf/lip across the enterocyte barrier and BBB via LfR‐mediated transcytosis. Next, the human colon carcinoma Caco‐2 cells were used as an enterocyte model to investigate the cellular uptake behavior of the SP‐Lf nanosystem. A transwell‐based Caco‐2 cell model was established to mimic the enterocyte barrier in vitro (Figure 3G). The transendothelial electrical resistance (TEER) value of cells remained unchanged at pre/post‐administration, implying the integrity of the monolayer (Figure S6A). CLSM images in Figure 3H showed that the fluorescence of FITC was mainly distributed above the monolayer. By contrast, the SP‐Lf/FITC group showed strong fluorescence of FITC within the Caco‐2 monolayer, revealing excellent enterocyte barrier penetration ability. For both the mucus model and enterocyte barrier model, SP‐Lf/lip nanoparticles collected from the basolateral medium exhibited intact morphology after crossing the barrier, as shown in Figure S7. Encouraged by these results, we moved on to assess the multiple‐barriers‐crossing efficiency of CBs‐based oral biotherapeutics in vivo. The mice were orally administered with FITC, CBs/FITC, and CBs‐Lf/FITC, respectively. The immunostaining micrographs of intestinal tissue in Figure 3I showed that the free FITC group was mainly distributed in the intestinal lumen. Compared with the CBs/FITC group, the CBs‐Lf/FITC group led to stronger fluorescence of FITC in the intestinal submucosa, confirming that the reassembled SP‐Lf/FITC significantly penetrated the intestinal mucus barrier and enterocyte barrier. For brain‐targeted drug delivery, crossing the BBB is challenging because the BBB, as the gateway to the brain, is one of the important self‐protection mechanisms of the host [31]. Surprisingly, a similar high cell penetration performance of SP‐Lf/FITC was also observed in murine brain capillary endothelial bEnd.3 cell‐based BBB model in vitro (Figure 3J,K; Figures S6B and S8). The enterocyte barrier and BBB‐crossing ability of SP‐Lf nanoparticles could be ascribed to the fact that Lf enhanced the enterocyte barrier and BBB permeability of the nanosystem via LfR‐mediated transcytosis.

Considering that the brain was the primary target of anti‐PD drug delivery, the distribution behavior of the formulation was evaluated in mice. According to the ex vivo fluorescence images of major tissues, Figure 3L showed that CBs‐Lf/Cy5 highly accumulated in the intestine in comparison with the Cy5 group, which could be assigned to the CBs‐mediated intestine targeting effect and the reassembled SP‐Lf/Cy5 formation in the intestine. Additionally, CBs‐Lf/Cy5 resulted in higher accumulation in the brain than the Cy5 group. The fluorescence signal of CBs‐Lf/Cy5 in the brain was 4.4 times higher than that of the Cy5 group (Figure S9). Moreover, the lip concentration in the brain was evaluated by LC‐MS technology, showing that the lip accumulation in the brain of CBs‐Lf/lip system was higher than that of the lip group (Figure S10). The above findings demonstrated the brain‐targeted ability of the CBs‐Lf system. The in vitro and in vivo barrier penetration experiments demonstrated the multiple‐barriers‐crossing ability of CBs‐Lf system. The CBs‐Lf could germinate in the intestine, and the SP shedding from CBs was reassembled into an SP‐Lf nanosystem, which penetrated the intestinal mucus barrier with the help of SP and crossed the enterocyte barrier as well as BBB via LfR‐mediated transcytosis to reach the brain (the primary target) (Figure 3M). Therefore, the probiotic spore‐based oral biotherapeutics broke through multiple barriers of oral brain‐targeted delivery. Next, the pharmacokinetics of CBs‐Lf/lip were evaluated (Figure S11 and Table S1). The time for CBs‐Lf/lip to reach maximum concentration (*t*_max_) was 2.27 ± 0.11 h, the mean residence time (MRT) and elimination half‐life time (*t*_1/2_) were 6.04 ± 0.09 h and 3.40 ± 0.18 h, respectively, which indicated a very promising pharmacokinetic profile for lip.

### 2.4 SP‐Lf/Lip Nanoparticles Reduced Oxidative Stress in Brain

Previous studies have indicated that oxidative stress constitutes a risk factor for neurodegeneration [32]. Oxidative stress induces an increase in reactive oxygen (ROS) and further lipid peroxidation. The end product of oxidation, malondialdehyde (MDA), destroys the structure and function of macromolecules, as well as aggravates membrane damage of neurons. Before examining the antioxidant effect of the reassembled nanosystem, the effect of SP‐Lf/lip on cell viability was assessed in neuron‐like rat pheochromocytoma cells (PC12) by using the CCK‐8 assay (Figure S12). No obvious cytotoxicity was found at the concentration range (lip: 0–50 nM), indicating the good biocompatibility of SP‐Lf/lip. Furthermore, the H_2_O_2_‐induced oxidative stress model of PC12 cells and dopaminergic neurotoxin 1‐methyl‐4‐phenyl‐1,2,3,6‐tetrahydropyridine (MPTP) induced PD mice were established. The increased fluorescence intensity of the ROS probe was found in H_2_O_2_‐treated cells as well as in the brain of MPTP‐treated mice (Figure 4A–D). Whereas other groups, especially SP‐Lf/lip, reduced ROS levels significantly.

**FIGURE 4:**
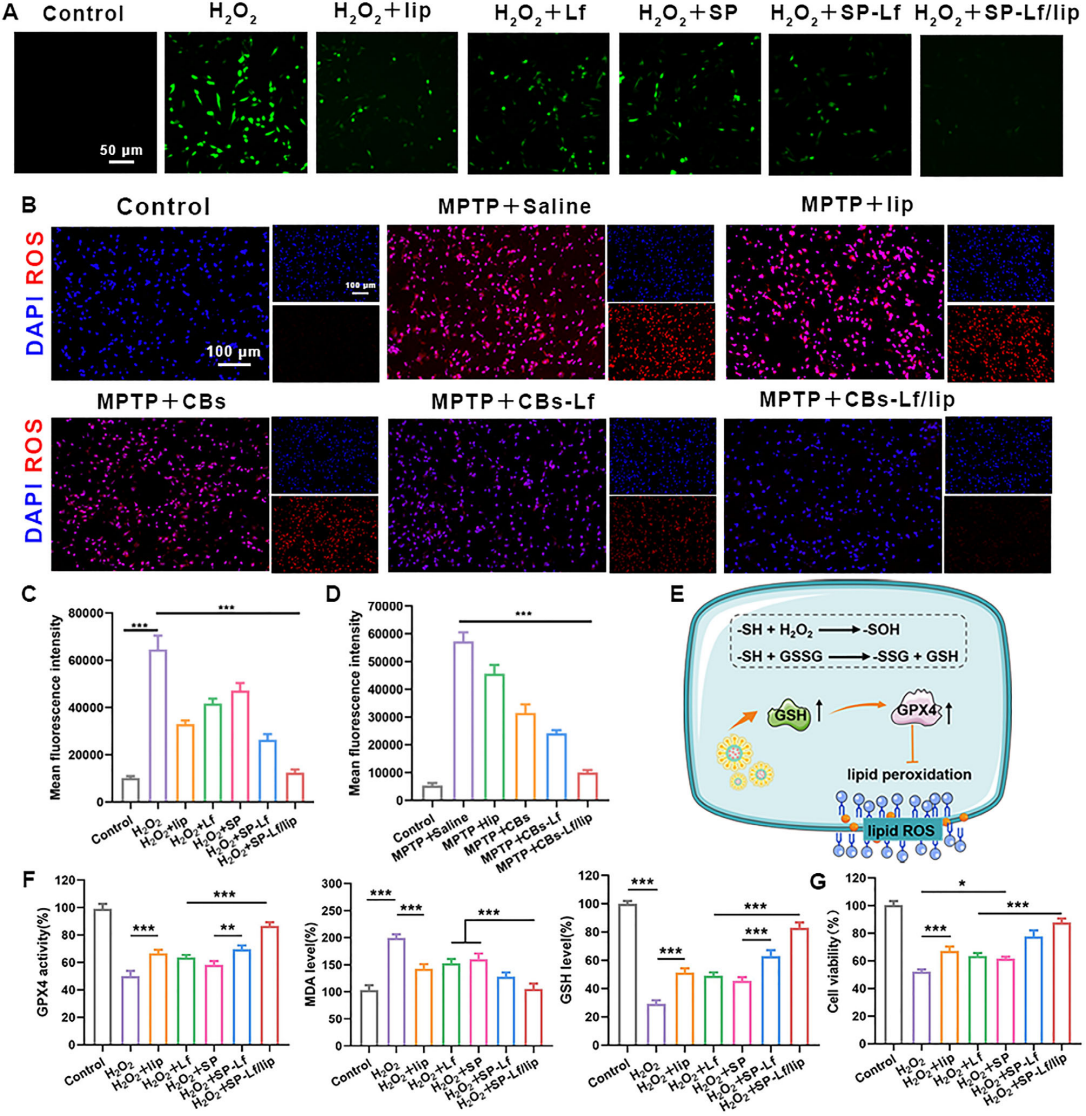
The SP‐Lf/lip and CBs‐Lf/lip decreased oxidative stress in neurons in vitro and in vivo, respectively. (A) ROS detection in PC12 cells after different treatments. (B) ROS detection in the substantia nigra of the brain. (C) Semi‐quantitative analysis of fluorescence of ROS probe in PC12 cells (*n *= 3). (D) Semi‐quantitative analysis of fluorescence of ROS probe in substantia nigra (*n *= 3). (E) Schematic diagram of the antioxidant ability of SP‐Lf/lip. The cellular oxidative stress is alleviated by SP‐Lf/lip due to efforts in many ways, such as the antioxidant activity of SP, the enhancement of glutathione peroxidase activity by Lf, and the inhibitory effect of lip on lipid peroxidation. (F) GPX4 activity, MDA, and GSH level of PC12 cells in the presence of H_2_O_2_ (*n *= 3). (G) The cell viability of neuron‐like PC12 cells in the presence of H_2_O_2_ (*n *= 6). Data were presented as means ± SD. *P < 0.05, **P < 0.01, ***P < 0.001.

In general, oxidative stress could activate the cellular antioxidant mechanism to counteract oxidative damage [33, 34]. Then the indicators of oxidative stress were measured (Figure 4F), with results showing that SP‐Lf/lip increased the activity of glutathione peroxidase 4 (GPX4, a key antioxidant enzyme) and the expression of glutathione (GSH, an antioxidant defense substance) while decreasing the level of MDA (the indicator of lipid peroxidation). The mechanism of the antioxidant property of SP‐Lf/lip might be involved in many aspects (Figure 4E): (1) The sulfhydryl groups abundant in SP tended to lose electrons in reactions, which could directly scavenge electrophilic reagents such as ROS and H_2_O_2_. The sulfhydryl groups also reacted with oxidized glutathione (GSSG) to produce GSH through thiol/disulphide exchange. (2) Lf could directly scavenge ROS and enhance the activity of antioxidant enzymes such as GPX4 [35]. 3) The lip as a lipid peroxidation inhibitor terminated free radical chain reactions, as well as promoted the GSH level and GPX4 activity to reduce oxidative stress [36]. Furthermore, the PC12 cell viability was assessed after H_2_O_2_ treatment. Figure 4G showed that H_2_O_2_ treatment resulted in a reduction in cell viability. However, the lip, Lf, SP, SP‐Lf, and SP‐Lf/lip treatments restored cell viability to varying degrees. These results suggested that the nanosystem was able to resist H_2_O_2_‐induced oxidative stress for neuroprotection. Overall, the CBs‐Lf/lip derived reassembled SP‐Lf/lip with brain‐targeted effect maintained the redox balance for neuroprotection with the efforts in many ways, which was expected to improve anti‐PD effect.

### 2.5 Therapeutic Evaluation of CBs‐Lf/Lip in PD Mice

PD is characterized by motor dysfunction and memory impairment due to dopaminergic neurons loss in the substantia nigra and striatum (Figure 5A) [1, 2]. Then, the dopaminergic neurotoxin MPTP administration was used to induce PD‐like symptoms in mice by simulating dopaminergic neuron degeneration. The PD mice were orally administrated with different formulations each day for 3 weeks (Figure 5B). Saline‐treated normal mice, saline‐treated PD mice, and clinical anti‐PD drug levodopa (L‐DOPA) treated PD mice were used as blank control, model control, and positive control, respectively. After that, a rotarod experiment was carried out (Figure 5C). PD mice showed the shortest time on the rotating rod due to unsteady gait and tremors. By contrast, the CBs‐Lf/lip group persisted on the rod for a long time, recovering the motor coordination ability to control the mice group. Then the behavioral pattern of mice was further evaluated by the open field test (Figure 5D–F). The MPTP‐induced PD mice tended to stay in the corners and edges of the open field, with the moving paths following the wall‐to‐wall model. However, the lip, CBs, and CBs‐Lf‐treated mice displayed longer total distance traveled in the test, as well as longer motor distance in the arena center. CBs‐Lf/lip group increased the total central motor distance to over 90% compared with the MPTP group, demonstrating enhanced desire for exploration and better autonomous motor function. Of note, the behavioral characteristics of mice in CBs‐Lf/lip group were comparable to those of the L‐DOPA group in the open field test and rotarod experiment. These data indicated that CBs‐Lf/lip alleviated behavioral deficits and exerted a compelling therapeutic effect in PD mice.

**FIGURE 5:**
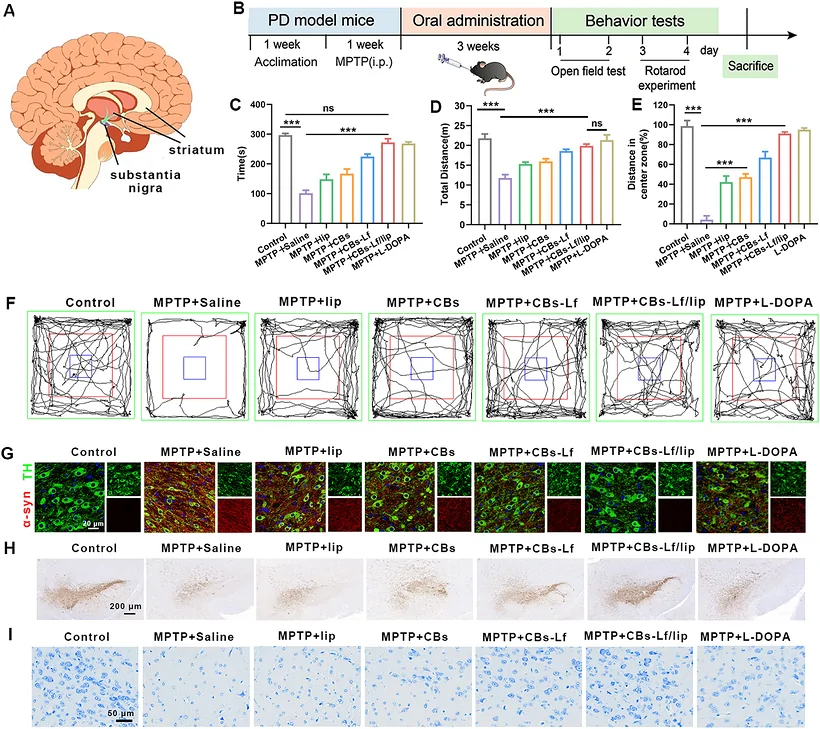
CBs‐Lf/lip attenuated motor deficits, increased TH expression, and protected neurons in PD mice. (A) Schematic diagram showing the lesion site (striatum and substantia nigra) in the brain of PD mice. (B) Schematic illustration of treatment regimens of PD mice. (C) In the rotarod experiment, the residence time of mice on the rotating rod was recorded (*n *= 6). (D) Total distance in the open field test was recorded (*n *= 6). (E) Total distance in arena center in open field test was recorded (*n *= 6). (F) Representative paths of mice in the open field test. (G) Representative immunofluorescence staining of α‐syn (red) and TH‐positive neurons (green) in substantia nigra. (H) Representative TH immunohistochemistry staining in substantia nigra. (I) Nissl staining of neurons in substantia nigra. Data were presented as means ± SD. *P < 0.05, **P < 0.01, ***P < 0.001.

Generally, many factors such as oxidative stress and gut microbiota dysbiosis lead to the abnormal aggregation of α‐synuclein (α‐syn) in the brain, which accelerates tyrosine hydroxylase (TH) positive dopaminergic neurons degeneration and reflects the severity of PD [37]. Figure 5G showed that CBs‐Lf/lip treatment also reversed the high expression and aggregation of α‐syn induced by MPTP. The TH immunohistochemistry staining in the substantia nigra showed a marked loss of dopaminergic neurons in the MPTP group (Figure 5H). On the contrary, CBs‐Lf/lip led to a substantial preservation of TH‐positive neurons. The situation of neuronal damage was further assessed by Nissl staining (Figure 5I). For the MPTP group, severe neuronal hypocellularity was observed. However, increased Nissl bodies were found after CBs‐Lf/lip treatment. The above results substantiated that CBs‐Lf/lip protected against dopamine neuron loss, enhanced neuronal density, and rescued PD symptoms. Of note, the L‐DOPA group alleviated behavioral deficits effectively, but it was less effective than CBs‐Lf/Lip in reversing neuronal loss. This could be attributed to the fact that L‐DOPA belongs to the dopaminergic replacement therapy; the pharmacological mechanism of L‐DOPA is to supplement the neurotransmitter dopamine. However, it does not intervene in the pathogenesis of PD, thereby treating the symptoms but not the root cause. In contrast, CBs‐Lf/Lip with neuroprotective effect significantly alleviated both behavioral deficits and dopaminergic neuron loss, displaying excellent therapeutic effect. To evaluate the biosafety of the biotherapeutic agent, the hematoxylin/eosin (HE) staining of major tissues was carried out (Figure S13). Results showed there was no histopathological lesion in each group. In addition, neither abnormalities in body weight nor biochemical indicator changes were found in each group (Figure S14A,B). Figure S14C displays that MPTP increased the expression of inflammatory cytokines (TNF‐α, IL‐6, and IL‐1β) in serum. However, CBs‐Lf/lip reduced the level of inflammatory cytokines, which was close to that of normal mice. The above results demonstrated that CBs‐Lf/lip had good biocompatibility.

### 2.6 Analysis of Gut Microbiota

The satisfactory therapeutic effects encouraged us to elucidate the therapeutic mechanism at the gut microbiota level. The feces of mice were collected and analyzed by 16S rDNA identification. Firstly, the flat Pan/Core species curve indicated the number of sequencing samples was sufficient for further species analysis (Figure S15A). Compared with normal mice, the PD mice led to a reduction in gut microbiota richness and diversity due to gut microbiota dysbiosis (Figure 6A). However, the abundance and diversity of microbiota were obviously improved after CBs‐Lf/lip treatment, as indicated by the improved operational taxonomic units (OTUs) (Figure S15B) and alpha diversity analysis (Figure 6A). To intuitively reflect the similarity and overlap of species, the Venn diagram of OTUs was analyzed (Figure 6B). It was found that 191 OTUs were shared by all groups, while the unique OTUs in saline, lip, CBs, CBs‐Lf, and CBs‐Lf/lip were 4, 13, 15, 22, and 26, respectively. These findings implied that different treatments changed the composition of microbiota. To analyze the similarity and difference in microbiota composition, beta‐diversity of microbial community was evaluated. In a non‐metric multidimensional scaling (NMDS) plot (Figure 6C), CBs‐Lf/lip displayed a centralized clustering of microbiota composition, which was closer to that of the normal mice group.

**FIGURE 6:**
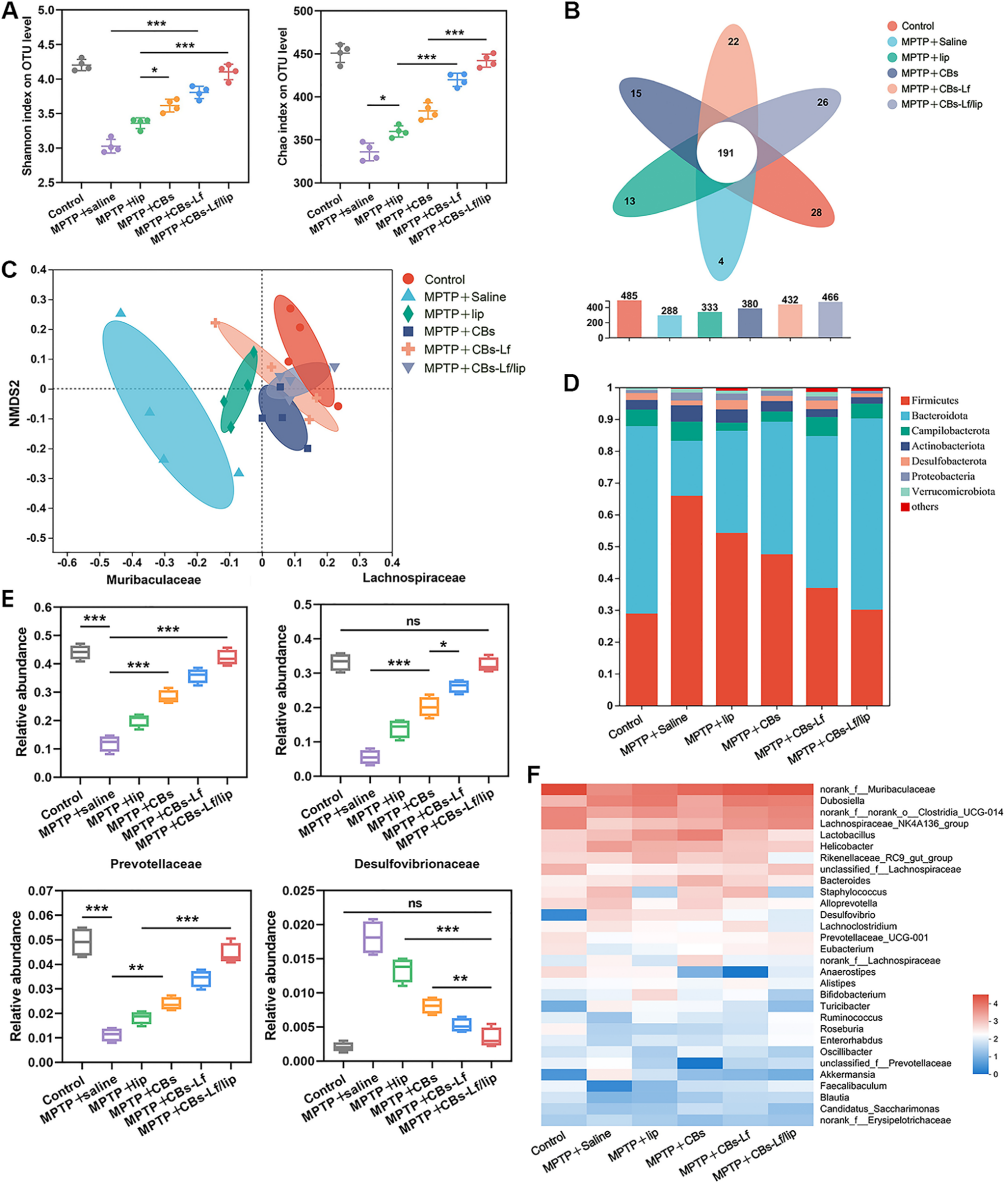
CBs‐Lf/lip regulated the gut microbiome in PD mice. The feces were collected and analyzed by 16S rDNA identification. (A) Alpha diversity of microbial community was evaluated by Shannon and Chao indices (*n *= 4). (B) Venn diagram of unique and shared OTUs. (C) Beta‐diversity of microbial community was evaluated by NMDS analysis. (D) Relative abundance of gut microbiome at the phylum level. (E) Relative abundance of PD‐related microbiome (Muribaculaceae, Lachnospiraceae, Prevotellaceae, Desulfovibrionaceae) on family level (*n *= 4). (F) Clustering heat map of species abundance on the genus level. Data were presented as means ± SD (*n *= 4). *P < 0.05, **P < 0.01, ***P < 0.001.

The relative abundances of gut microbiome on the phylum and family level were analyzed (Figure 6D,E), which visually showed that the treatments decreased the abundance of *Desulfovibrionaceae* (known to be associated with the development of PD), and increased the abundance of probiotics (e.g., *Muribaculaceae*, *Lachnospiraceae*, *Prevotellaceae*). Further analysis of species abundance on the genus level in Figure 6F showed that there were significant differences in microbial composition between the PD mouse group and the normal mice group. For example, *Desulfovibrio*, as the dominant microbiota, promoted the abnormal aggregation of α‐syn in the intestine, further exacerbating the pathological symptoms of PD [38]. In contrast, the CBs‐Lf/lip treatment decreased the relative abundance of species associated with the occurrence and development of PD, while increasing the relative abundance of probiotics (e.g., *Prevotellaceae_UCG‐001*, *Roseburia*, *Blautia*, *Faecalibacterium*) to promote the secretion of SCFAs. Collectively, these results provided strong evidence that the CBs Lf/lip treatment increased the abundance of probiotics and inhibited the colonization of potential pathogens in the gut, which suggested that our oral therapeutic agent reshaped gut microbiota and restored intestinal homeostasis.

### 2.7 CBs‐Lf/Lip Modulated Gut‐Brain Axis in PD Mice

In‐depth research has found that the influence of the gut microbiota is not limited to the intestine, but also to the central nervous system [39, 40, 41]. The microbiota participates in and regulates brain function via the microbiome‐gut‐brain axis (endocrine pathway, immune system, nerve pathway) [13, 14]. Based on the reshaping effect of CBs‐Lf/lip on gut microbiota, the mechanism of gut microbiota on neuropathology was further investigated. The level of butyric acid (a kind of SCFA) in feces, as the dominant neuro‐active metabolite secreted by *C. butyricum* was detected (Figure 7A). The butyric acid level in PD mice decreased significantly compared with that of normal mice, while different treatments improved the butyric acid level to varying degrees. These results implied that the gut microbiota and its metabolites had a non‐negligible impact on the occurrence and development of PD. According to the literature, SCFAs act as intermediaries in microbiome‐gut‐brain crosstalk which are associated with the reduction of neuroinflammation [13, 14]. As shown in Figure 7B, CBs‐Lf/lip reduced the expression of pro‐inflammatory cytokines such as TNF‐α and IL‐1β in the substantia nigra. Generally, SCFAs bind to free fatty acid receptor 2 on intestinal L cells, thereby promoting the secretion of neuroprotective GLP‐1 to activate the GLP‐1/GLP‐1R signaling (Figure 7C). Immunofluorescence staining of GLP‐1 in Figure 7D showed that CBs‐Lf/lip improved the GLP‐1 level, which was close to that of normal mice. The GLP‐1 receptor (GLP‐1R) is widely distributed in the central nervous system, such as in the substantia nigra associated with PD lesions. Then, GLP‐1 entered the brain through the endocrine pathway to activate the GLP‐1/GLP‐1R signaling, which laid the foundation for anti‐inflammation and neuroprotection.

**FIGURE 7:**
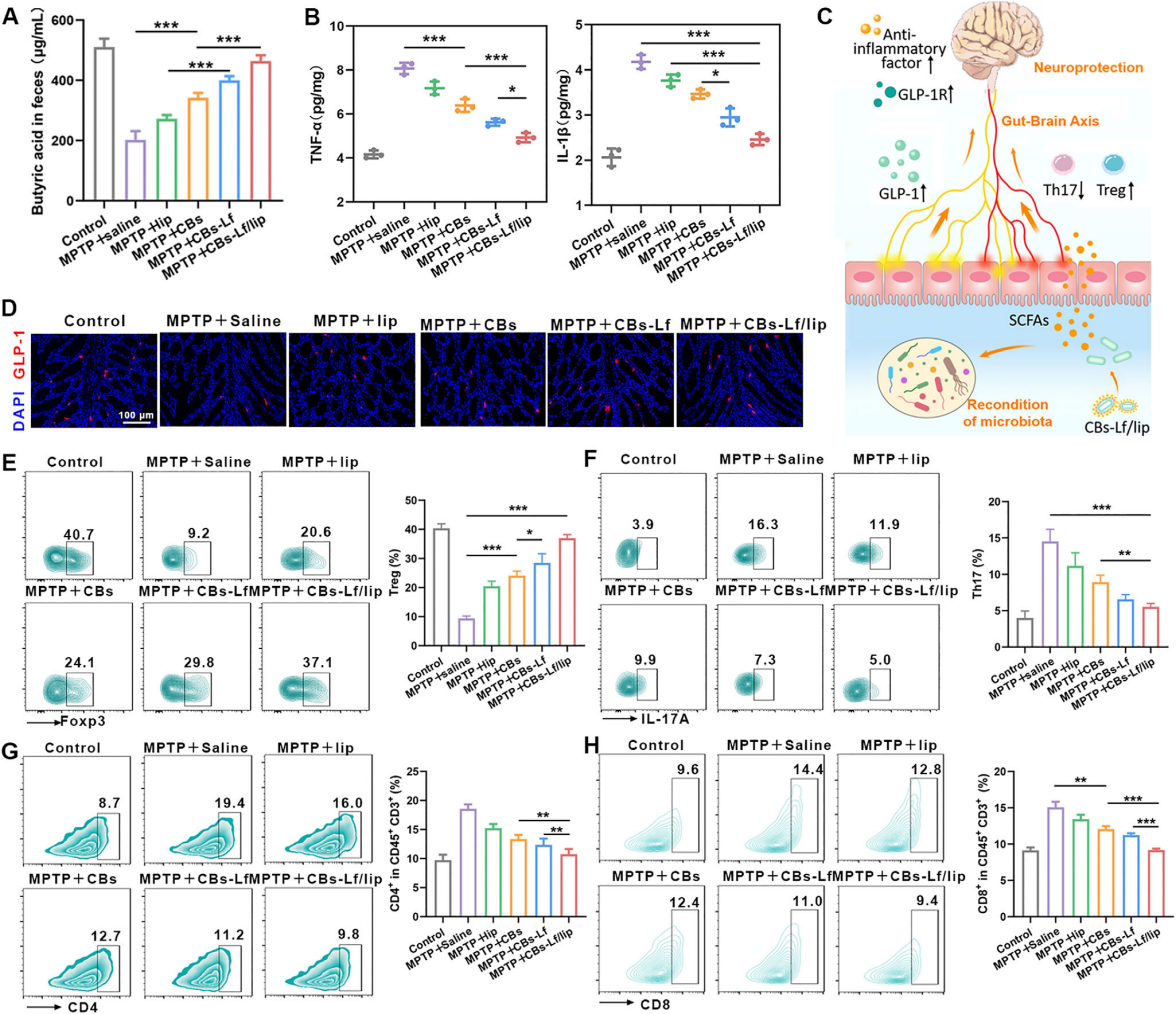
CBs‐Lf/lip regulated gut‐brain axis in PD mice. (A) Butyric acid level detection in feces (*n *= 3). (B) TNF‐α and IL‐1β level detection in substantia nigra via ELISA analysis (*n *= 3). (C) Schematic diagram of the gut‐brain interaction regulated by CBs‐Lf/lip. CBs‐Lf/lip germinate into *Clostridium butyricum* and colonize the intestine (the secondary target). Their secreted neuroactive SCFAs restore intestinal immune homeostasis as well as promote intestinal GLP‐1 secretion, thereby affecting neurophysiology and reducing neuroinflammation for neuroprotection through gut‐brain signaling (endocrine pathway, immune system, and nerve pathway). (D) Representative immunofluorescence staining of GLP‐1 (red) in colon tissue. (E) Flow cytometry plots and quantitative analysis of Treg cells in colon tissue (*n *= 3). (F) Flow cytometry plots and quantitative analysis of Th17 cells in colon tissue (*n *= 3). (G) Flow cytometry plots and quantitative analysis of CD4^+^ T cells in brain (*n *= 3). (H) Flow cytometry plots and quantitative analysis of CD8^+^ T cells in brain (*n *= 3). Data were presented as means ± SD. *P < 0.05, **P < 0.01, ***P < 0.001.

The gut microbiota and its metabolites are able to interact with the host to alter intestinal immune homeostasis, the gut microbiota dysbiosis and further intestinal immune activation may be potential drivers of PD inflammation [42, 43]. To investigate the effect of CBs‐Lf/lip on intestinal immunity, flow cytometry analysis of immune cell populations was performed. Compared to normal mice, the abundance of regulatory T cells (Tregs) in PD mice decreased (Figure 7E). The frequencies of Tregs in lip, CBs, CBs‐Lf and CBs‐Lf/lip groups were higher than those of PD mice. Meanwhile, the decreased T helper cell 17 (Th17) was observed after different treatments (Figure 7F). The above results demonstrated that the SCFAs enhanced by CBs‐Lf/lip increased the differentiation of naive T cells into Treg cells and inhibited the inflammatory Th17 polarization, which shaped the Th17/Treg balance and maintained intestinal immune homeostasis. Furthermore, MPTP increased the infiltration of CD4^+^ T cells and CD8^+^ T cells into the brain compared to the control group (Figure 7G,H). However, CBs‐Lf/lip suppressed the infiltration of these immune cells. In conclusion, the excellent anti‐inflammatory effect of CBs‐Lf/lip may be ascribed to the following reasons: (1) SCFAs not only maintained intestinal immune balance to reduce the infiltration of peripheral immune cells into the brain, but also could be absorbed by colonocytes and then directly cross the BBB via monocarboxylate transporters to regulate the immune microenvironment of the brain, thereby inhibiting neuroinflammation. (2) The reassembled SP‐Lf/lip also reduced oxidative stress and reduced neuroinflammation in the brain. Taken together, the CBs‐Lf/lip germinated into *C. butyricum* and colonized the intestine (the secondary target) to modulate the gut‐brain axis for neuroprotection, which compensated for the shortage of traditional PD treatment that only intervened in brain function.

## 3 Discussion

Most research on PD delivery systems focuses on intravenous and intraventricular administration, which limits the long‐term PD management [1, 2]. In contrast, non‐invasive oral brain‐targeted drug delivery possesses advantages of convenience and safety; however, it has been rarely studied. This is mainly due to the inevitable multiple barriers in vivo that significantly hinder the brain accumulation of oral formulations. This work focuses on developing an oral anti‐PD system to address the challenges in oral brain‐targeted delivery. The CBs‐Lf/lip system with multiple‐barriers‐crossing ability dual‐regulates gut‐brain, holding promise for providing a convenient, safe, and comprehensive therapeutic approach for PD. The dual‐targeting/regulation capability of CBs‐Lf/lip relies on the synergistic effects of two functional components: the CBs‐mediated gut targeting/regulation effect and SP‐Lf/lip mediated brain targeting/regulation effect. To more comprehensively evaluate the effectiveness of the CBs‐Lf/lip system, we included the CBs group (single gut targeting/regulation) and the lip group (single brain regulation) to compare with the CBs‐Lf/lip group (dual gut‐brain targeting/regulation). Results showed that CBs‐Lf/lip displayed the best gut‐brain regulation capacity and behavioral characteristics. Therefore, the synergy of dual targeting/regulation of both gut and brain is the key to anti‐PD efficacy of CBs‐Lf/lip. In terms of anti‐PD efficacy, SP‐Lf/lip and CBs form complementary and synergistic relationships at different stages of PD treatment. After 24 h of administration, SP‐Lf/lip can quickly reach the brain to reduce oxidative stress. It acts as a commando in the early stage of PD treatment, filling the treatment gap before spore takes effect. By contrast, CBs need multiple administrations and a longer period (usually >14 days) to complete the cascade reaction of intestinal colonization, microbiota remodeling, and gut‐brain axis signaling [44]. CBs act slowly but serve as a lasting adjunctive pathway, providing long‐term anti‐inflammatory and neurotrophic support to the brain. Collectively, CBs‐Lf/lip mediated the dynamic adaptation of dual gut‐brain regulation, comprehensively covering the progressive pathological processes of PD, providing a dual‐pronged synergistic platform for PD treatment.

The CBs‐Lf/lip exhibit excellent neuroprotective effect, as is evidenced by TH‐positive neuron preservation (Figure 5G,H) and maintenance of neuronal structure according to the Nissl staining (Figure 5I). The gut‐brain axis modulation and the reduced oxidative stress are responsible for the neuroprotection. In terms of intestinal parameters, the gut microbiota secreted neuro‐active SCFAs activate the GLP‐1/GLP‐1R signaling (Figure 7D), which exerts neuroprotective effects to promote neuronal survival [45]. Moreover, the mechanism of the immune pathway of the gut‐brain axis is also investigated. CBs‐Lf/lip shape the intestinal Th17/Treg balance, suppress the infiltration of peripheral immune cells into the brain (Figure 7E–H), and reduce the expression of pro‐inflammatory cytokines in the substantia nigra (Figure 7B). Such excellent anti‐inflammatory effect of CBs‐Lf/lip is conducive to their neuroprotective effect. In addition, the degree of oxidative stress in the brain is intrinsically tied to neuroprotection. The in vitro cellular experiments have directly demonstrated a negative correlation between oxidative stress and neuronal viability, as is evidenced by measurements of ROS, MDA, and GSH levels, as well as GPX4 activity (Figure 4A,F,G). The in vivo experiments show that CBs‐Lf/lip reduces ROS levels significantly compared to the MPTP group (Figure 4B), thereby mitigating oxidative damage to dopaminergic neurons. The above results provide complete evidence for the neuroprotective mechanism of CBs‐Lf/lip.

Despite the excellent anti‐PD efficacy of CBs‐Lf/lip, there are still limitations such as the lack of long‐term efficacy evaluation and non‐motor assessment, which will be addressed in future research. Probiotic spores have unique advantages in oral delivery; however, their clinical application still faces many challenges: (1) The variability in spore germination. Variations in the individual intestinal microenvironment can lead to inconsistent spore germination rates. Moreover, the germination sites of spores under normal and pathological conditions are uncontrollable. (2) The stability differences of spores under different physiological conditions. Although spores are acid‐resistant, different strains have varying abilities to withstand bile salts, and certain spores may become inactive at high levels of bile salts. Digestive enzymes in the intestine also affect the colonization of spores. 3) Challenges in large‐scale production and quality control. Usually, the industrial spore yield (70%–80%) is lower than that of the laboratory (>90%). The purification of spores is more challenging and costly than that of probiotic cells. Currently, researchers are actively addressing these issues by delving into germination mechanisms, optimizing fermentation processes, and developing smart delivery systems, advancing spore‐based probiotic therapies toward clinical applications.

## 4 Conclusion

The preferred route of administration is a critical factor in the delivery system development. However, a multitude of obstacles to oral brain‐targeted drug delivery make the development of an oral system extremely challenging. On the basis of our previous research on the reassembly of probiotic spores into nanoparticles, this study finds additional excellent characteristics of probiotic spores, including the function of reassembling into a nanosystem with multiple‐barrier‐crossing efficiency and antioxidant properties, gut‐brain communication, and so on. In light of these, probiotic spores as an oral delivery carrier hold significant value for gut‐brain dual‐regulation in PD treatment. This work shows that the spores germinate into probiotics in the intestine to affect neurophysiology via the gut‐brain axis. The SP shedding from spores is reassembled into nanosystems, which cross multiple barriers (chemical barrier, intestinal mucus barrier, enterocyte barrier, and BBB) to reach the brain and further reduce oxidative stress. Data show the designed oral live biotherapeutic agent reduces PD‐associated neuropathology and alleviates motor and memory dysfunction in mice. Overall, this work emphasizes a comprehensive and promising technique for gut‐brain dual‐regulation, addresses the limitations of traditional strategies that only intervene in brain function, and may be further extended to treat other central nervous system diseases (Alzheimer's disease, ischemic stroke, etc).

## Author Contributions

**Qianhua Feng**: conceptualization, writing – original draft, funding acquisition. **Yuying Mei**: methodology, investigation, data curation, formal analysis, software. **Jia Zhang**: investigation, data curation. **Liying Sun**: investigation, data curation. **Bingqian Si**: methodology, software. **Huimin Zhou**: methodology. **Huifang Xiao**: formal analysis; funding acquisition. **Runhan Liu**: writing – review and editing. **Jieyun Zhang**: investigation. **Lei Wang**: conceptualization; writing – review and editing, funding acquisition.

## Conflicts of Interest

The authors declare no conflicts of interest.

## Ethics Statement

All procedures associated with animals were performed according to the National Institutes of Health Guide for the Care and Use of Laboratory Animals and were approved by the Animal Ethical Committee of Zhengzhou University (yxyllsc20240086).

## Supporting information




**Supporting File**: exp270221‐sup‐0001‐SuppMat.doc.

## Data Availability

The data that support the findings of this study are available from the corresponding author upon reasonable request.
