# Supplementary material for: Probiotic Spore Inspired Oral Drug Delivery System With Multiple‐Barriers‐Crossing Ability Modulates Gut‐Brain Axis for Parkinson's Disease Therapy
Source: Exploration (Beijing). 2026 Sep 9;6(5):70221. doi: 10.1002/exp2.70221 (PMC13555365; doi:10.1002/exp2.70221)
Supplement: Supplementary file 1 [file EXP2-6-70221-s001.doc]

Supporting Information

**Probiotic Spore Inspired Oral Drug Delivery System with Multiple-Barriers-Crossing Ability Modulates Gut-Brain Axis for Parkinson’s Disease Therapy**

**Materials and methods**

*Materials*: Lactoferrin (Lf) was obtained from Aladdin Reagent (Shanghai, China). Liproxstatin-1(lip) was procured from Glpbio (USA). N-(3-dimethylamino propyl-N’-ethylcarbodiimide) (EDC), N-Hydroxysuccinimide (NHS), 1-Methyl-4-phenyl-1,2,3,6-tetrahydropyridine (MPTP) were procured from Sigma-Aldrich. Other reagents were standard Sinopharm Chemical Reagent (Shanghai, China).

*Preparation and characterization of CBs-Lf/lip*: Lf (20 mg), EDC (64 mg) and NHS (30 mg) were dissolved in PBS and mixed, then stirred for 1 h to activate the carboxyl groups of Lf. After that, CBs (10 mg) were added into the mixture and stirred for 24 h. After centrifugation, the resulted CBs-Lf were suspended in PBS and mixed with 2 mL of ethanol containing lip (10 mg). After reaction for 24 h, the product was washed and concentrated to obtain CBs-Lf/lip. Zetasizer Nano ZS-90 instrument was used to detect the zeta potential of samples. The drug loading capacity was characterized by Ultraviolet-visible (UV/Vis) spectrum (L-2489).

*Growth behavior of CBs-Lf/lip*: To investigate the effects of Lf and lip on spore germination and growth, the growth behavior of bacteria was assessed by OD600 (optical density at 600 nm) detection as well as plate counting method. For OD600 detection method, the samples (CBs, CBs-Lf and CBs-Lf/lip) were diluted in brain heart infusion (BHI) broth and cultured under anaerobic condition for 24 h at 37 ℃ with gently shaking, respectively. Then 200 μL of sample was collected and the OD600 value was recorded with a microplate reader. For plate counting method, the sample solutions of CBs, CBs-Lf and CBs-Lf/lip were spread on BHI agar plates after dilution, respectively. The culture plates were incubated under anaerobic condition for 24 h and then the bacterial colonies were counted.

*The ability of CBs-Lf/lip to overcome biochemical barrier*: The CBs-Lf/lip were cultured in PBS and simulated gastric fluid for 2 h, respectively. Then the samples were collected after centrifugation. The morphology of spore was visualized by TEM. In addition, CBs-Lf/lip was placed in dialysis bag (MWCO 1000) and incubated in simulated gastric fluid at 37 ℃ with gently shaking. The leaked lip samples from CBs-Lf/lip to simulated gastric fluid were removed at predetermined time intervals. The concentration of lip was quantified spectrophotometrically and the drug leakage rate was calculated.

*The preparation and characterization of reassembled SP-Lf/lip in vitro*: CBs-Lf/lip were inoculated into BHI broth and cultured under anaerobic condition at 37 ℃ for 24 h. As CBs germinated, the spore protein shell (SP), Lf and lip shed from the surface of CBs. The solution was centrifuged at 8000 rpm for 10 min. The supernatant was collected and centrifuged at 20000 rpm for 15 min. The precipitation was collected to obtain SP-Lf/lip. For SP-Lf preparation, CBs-Lf were cultured in BHI broth under anaerobic condition and centrifuged according to the method of SP-Lf/lip synthesis. The content of sulfhydryl group in SP was detected by sulfhydryl assay kit.

*Bio-TEM*: After oral administration of CBs-Lf/lip for 6 h, mice were sacrificed. The intestinal tissues were removed, fixed with osmic acid (1%) at 4 °C for 3 h, and then dehydrated with ethanol (50%, 70%, 80%, 90%, 100%) gradually. The tissues were embedded in resin, stained with uranyl acetate and measured by TEM.

*The multiple-barriers-crossing ability of CBs-Lf and SP-Lf*: To evaluate intestinal mucus barrier penetration ability of SP-Lf nanoparticles, a transwell model of mucus was established. The mucin was dissolved in PBS and then placed uniformly on the apical chamber. PBS was added to the basolateral chamber. Next, Lf/lip and SP-Lf/lip were introduced to the apical insert. The transwell plate was incubated at 37 ℃ with gently shaking. The culture medium in basolateral chamber was removed and centrifugated to obtain the nanoparticles, and the penetrated nanoparticles containing lip were quantified spectrophotometrically. The apparent permeability coefficient (P_app_) of lip was calculated by the following formulas: P_app_ = dQ/dT*1/(A*C_0_), where the dQ/dT presented the diffusion rate of lip from apical chamber to basolateral chamber, A presented area of polycarbonate film, C_0_ presented initial concentration of lip in apical chamber.

For the *in vitro* enterocyte barrier permeability experiment, transwell system was used to simulate the enterocyte barrier model. Human colon carcinoma Caco-2 cells were seeded in apical chamber. After culturing for 21 days, the monolayer was reasonable to simulate the morphology and activity of enterocyte barrier. The transendothelial electrical resistance (TEER) value above 450 Ω·cm^2^ was used for further experiment. Medium containing FITC or SP-Lf/FITC was added into apical chamber under dark, respectively. After 3 h, the TEER value was detected to examine the effect of SP-Lf/FITC on the integrity of monolayer. Then cells were visualized with CLSM.

For the *in vitro* BBB permeability experiment, murine brain capillary endothelial bEnd.3 cells were seeded in apical chamber of transwell. After 14 days, the monolayer was reasonable to simulate the morphology and activity of BBB. The TEER value above 200 Ω·cm^2^ confirmed the structural integrity. Media containing FITC or SP-Lf/FITC were added into apical chamber under dark and co-incubated for 3 h, respectively. After that, TEER value was measured to investigate the effect of SP-Lf/FITC on the integrity of monolayer. bEnd.3 cells were collected and stained with DAPI. CLSM was used to analyze the fluorescence.

To assess the *in vivo* intestinal mucus barrier and enterocyte barrier penetration ability of CBs-Lf, mice were orally administrated with FITC, CBs/FITC and CBs-Lf/FITC, respectively. After treatments for 3 h, intestinal tissues were harvested. The mucin was stained with iFluor^TM^ 647-conjugated wheat germ agglutinin, and the fresh-frozen sections of intestinal tissue were further stained with DAPI. These sections were fixed on the glass slide and observed under CLSM.

*Fluorescence imaging in vivo*: Mice received oral administration of Cy5 and CBs-Lf/Cy5, respectively. At 24 h post-injection, mice were sacrificed. Major organs were harvested and washed. The fluorescence images were captured with IVIS imaging system (λ_ex_ = 640 nm, λ_em_ = 670 nm).

*Cell viability assay*: To measure the biocompatibility of SP-Lf/lip, rat pheochromocytoma cells (PC12) were seeded in 96-well plates at 8×10^3^ per well and cultured overnight. Then medium containing SP-Lf/lip was used to replace the old medium. After 24 h, cells were incubated with cell counting kit-8 (CCK-8), and the optical density at 450 nm was detected by microplate reader to calculate the cell viability. To assess the effect of SP-Lf/lip on cell viability in the presence of H_2_O_2_, PC12 cells were seeded in 96-well plates at 8×10^3^ per well and cultured overnight. Then cells were cultured with PBS, lip, Lf, SP, SP-Lf and SP-Lf/lip (lip: 20 nM) in the presence of H_2_O_2_ (400 μM) for 24 h, respectively. The cells viability was assessed by using CCK-8 assay. In addition, the morphology of cells was captured by digital camera.

*The oxidative stress assessment in vitro*: The biomarkers of oxidative damage such as GPX4, GSH and MDA were quantified. Briefly, PC12 cells were treated with medium containing PBS, lip, Lf, SP, SP-Lf and SP-Lf/lip (lip: 20 nM) in the presence of H_2_O_2_ (400 μM), respectively. After culturing for 24 h, cells were harvested with a cell scraper, homogenized, and further centrifuged to obtain the supernatants. At last, the contents of GPX4, GSH and MDA were detected by relevant reagent kits, respectively.

For the detection of ROS, PC12 cells were treated with PBS, lip, Lf, SP, SP-Lf and SP-Lf/lip (lip: 20 nM) in the presence of H_2_O_2_ (400 μM) for 6 h, respectively. Next, cells were washed and incubated with DCFH-DA probe under dark for 30 min. The fluorescence images was captured by CLSM.

*Establishment of PD model and treatment*: 6-week-old male C57BL/6 mice were kept at 23 ± 2 ℃ with free access to water and food. Mice were divided into six groups randomly (*n* = 6): negative control group (normal mice), MPTP + Saline group (PD mice model), MPTP + lip group, MPTP + CBs group, MPTP + CBs-Lf group, MPTP + CBs-Lf/lip group, MPTP + L-DOPA group. Except for control group, mice were intra-peritoneally injected with MPTP (30 mg kg^-1^) each day for 7 days to establish PD model. And the control group received saline for 7 days. Simultaneously, after the end of the last day of MPTP injection, mice were orally administrated with the above formulations (lip: 6 mg kg^-1^, CBs: 1×10^9^ CFUs mL^-1^, 100 μL, L-DOPA: 40 mg kg^-1^) each day for 3 weeks. After that, behavioral tests were carried out.

*Behavioral tests*: For open field test, the square open field was divided into 16 squares with a white bottom and black walls. The 4 squares in the middle of the field were called the arena center, and the surrounding 12 squares were called the arena periphery. When the test began, mouse was placed in the open field and allowed to move randomly for 5 min. The movement track, total distance in arena center were recorded and analyzed by SMART 3.0 system. After the experiment, the open field was wiped with alcohol to avoid the odor of the previous mouse from affecting the experimental results of the next mouse.

For rotarod experiment, the fatigue rotating rod with a diameter of 3 cm and a certain roughness on the surface was used. Mice were pre-trained on the rod at a constant speed of 9 rpm for 10 min. The formal test was initiated by putting on mice on the rod at a steady growth rate of 5-40 rpm. Three independent experiments were performed on each mouse, with an interval of 30 minutes between each experiment. The residence time of mice on the rotating rod was recorded to evaluate the motor coordination of mice.

*Histological analysis*: For immunofluorescence analysis, the brains were harvested, fixed and embedded in paraffin. Primary antibodies such as anti-α-synuclein (anti-α-syn, Servicebio, GB11404) anti-tyrosine hydroxylase (anti-TH, Servicebio, GB11181) were incubated with the above sections at 4 ℃ overnight. Then the appropriate secondary antibodies were applied at room temperature for 1 h, followed by incubation with fluorescein tyramide. The fluorescence images were recorded under CLSM. Furthermore, the colon tissues were cut into sections according to the method of preparation of brain sections. These sections were incubated with anti-glucagon-like peptide-1 (anti-GLP-1, Abcam, ab133329) and then fluorescent secondary antibody. Then sections were examined under CLSM.

For immunohistochemistry analysis, coronal and sagittal sections of brains were prepared according to the above methods, respectively. Then sections were incubated with anti-TH and then secondary antibody. Using diaminobenzidine as the chromogen, TH-positive neurons with brown granules in striatum and substantia nigra were visualized under light microscopy. For Nissl staining, brain sections were washed and Nissl body was stained with 0.1% cresyl violet. The histological assessment was analyzed under microscope.

*Enzyme-linked immunosorbent assay (ELISA)*: The harvested substantia nigra was homogenized in cold PBS. The homogenate was centrifuged at 4 ℃. The supernatant was collected, the TNF-α and IL-1β levels in substantia nigra were detected by using ELISA kits. And the optical density at 450 nm was recorded by microplate reader.

*Flow cytometry analysis*: The harvested colon tissues were washed by PBS and chopped into pieces. To remove intestinal mucin, tissue fragments were placed in PBS containing dithiothreitol (1mM) and EDTA (5 mM) with gentle shaking at 37 ℃ for 30 min. Next, the tissues were digested with collagenase IV for 1 h with gentle shaking, then homogenized. The cells were counted and incubated with GolgiPlug protein transport inhibitor in 6-well plate for 6 h. Cells were stained with live/dead kit. Subsequently, cells were washed, resuspended in PBS containing fluorescence-labelled antibodies (anti-CD45, anti-CD3, anti-CD4, anti-Foxp3, anti-IL-17A). Flow cytometer (BD LSRII) was used for fluorescence activated cell sorting. The data was analyzed by FlowJo 10.6.2 software.

*Gut microbiota analysis*: Three days before mice were sacrificed, fecal pellets were collected into sterile tubes containing dry ice and stored at -80 ℃. 16S rDNA identification was conducted in Shanghai Majorbio Technology Co., Ltd (Wuhan, China). In brief, microbial genomic DNA was extract from fecal pellets and 16S rRNA gene amplification was applied on Illumina Miseq sequencing platform. Alpha diversity of microbial community was evaluated by shannon and chao indexes. The abundance of microbiota was used to assess the screen for differential species.

*Statistical analysis*: Data were presented as means ± SD. All data sets were analyzed by using GraphPad Prism6 software. The differences between two groups and multiple groups were analyzed by Student’s *t* test and one-way ANOVA, respectively. The level of significance was set at probabilities of **P* < 0.05, ***P* < 0.01 and ****P* < 0.001.

**Figure S1.** Growth study of CBs, CBs-Lf and CBs-Lf/lip by using OD600 recording after culturing spores in growth medium for 24 h (*n* = 3).

**Figure S2.** Drug leakage rate of CBs-Lf/lip in simulated gastric fluid (*n* = 3).

**Figure S3.** TEM images of CBs-Lf/lip after storage at 4 ℃ for 0 d, 7 d and 14 d, respectively.

**Figure S4.** Mechanism for the reassembly of SP-Lf nanoparticles (*n* = 6).

**Figure S5.** The LRP1 expression (green) in intestine (A) and brain (B).

**Figure S6.** The changes in transendothelial electrical resistance (TEER) values at pre/post-administration in (A) enterocyte barrier and (B) BBB transwell model (*n* = 3).

**Figure S7.** SEM images of SP-Lf/lip nanoparticles in basolateral medium in transwell-based mucus model (A) and enterocyte barrier model (B).

**Figure S8.** Semi-quantitative analysis of fluorescence of FITC in BBB model *in vitro* (*n* = 3).

**Figure S9.** Region-of-interest analysis of the averaged fluorescence intensity in each tissue (*n* = 3).

**Figure S10.** The lip concentration in brain following the oral administration of CBs-Lf/lip and lip in a dose equivalent to 6 mg kg^-1^ of lip at 24 h, respectively (*n*=3).

**Figure S11.** Plasma concentration-time curves of mice following the oral administration of CBs-Lf/lip in a dose equivalent to 60 mg kg^-1^ of lip (*n*=3).

**Figure S12.** Cell viability of neuron-like PC12 cells (*n* = 6).

**Figure S13.** H&E staining of major tissues.

**Figure S14.** A) Body weight of mice (*n* = 6). B) Blood biochemistry and hematology surveys of white blood cells (WBC), red blood cells (RBC), platelets (PLT), hemoglobin (HGB), liver function markers including alanine aminotransferase (ALT), aspartate aminotransferase (AST), renal function indicators including blood urea nitrogen (BUN) and creatinine (CREA) (*n* = 6). C) The expression of inflammatory cytokines TNF-α, IL-6 and IL-1β in serum (*n* = 4).

**Figure S15.** 16S rDNA analysis of gut microbiome. (A) Pan/core analysis. (B**)** OTU rank-abundance distribution curve in different groups.

**Table S1** Pharmacokinetic parameters of CBs-Lf/lip following oral administration (*n*=3).

| T_max_ (h) | C_max_ (μg mL^-1^) | AUC_0-t_ (μg mL^-1^*h) | MRT (h) | t_1/2_ (h) |
| --- | --- | --- | --- | --- |
| 2.27 ± 0.11 | 10.64 ± 0.39 | 71.82 ± 2.69 | 6.04 ± 0.09 | 3.40 ± 0.18 |
